# Supplementary material for: Stage-Dependent Activity and Pro-Chondrogenic Function of PI3K/AKT during Cartilage Neogenesis from Mesenchymal Stromal Cells
Source: Cells. 2022 Sep 23;11(19):2965. doi: 10.3390/cells11192965 (PMC9563299; doi:10.3390/cells11192965)
Supplement: Supplementary file 1 [file cells-11-02965-s001.zip › cells-1917252-supplementary.pdf]

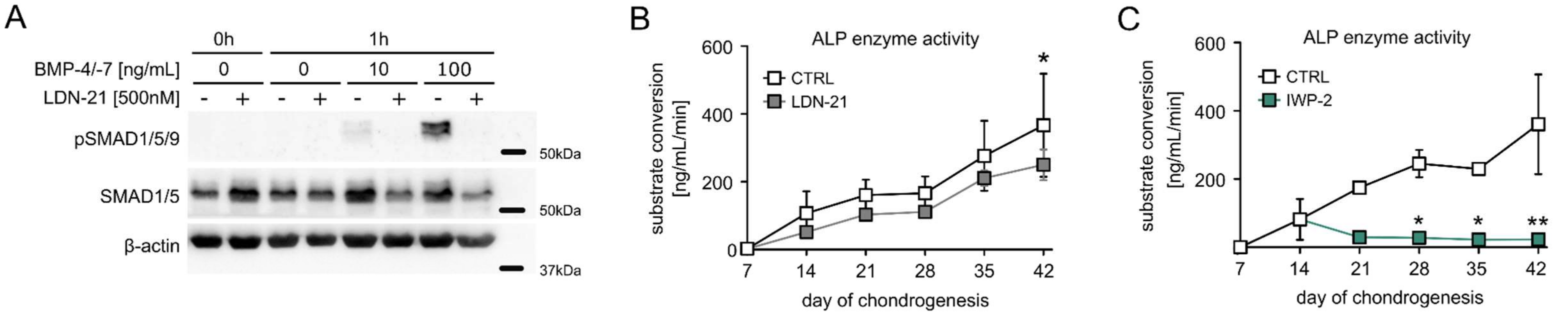

Supplemental Figure S1: Reduction of ALP enzyme activity during MSC chondrogenesis by the BMP inhibitor LDN-21 or the WNT inhibitor IWP-2. (**A**) MSC pellets were pre-treated on day 0 for 2 hours with LDN-21 or DMSO in chondrogenic medium, before 10ng/mL or 100ng/mL of each BMP-4 and BMP-7 were added for 1 hour in the presence or absence of LDN-21. Protein levels of phosphorylated SMAD1/5/9 were detected by Western blot using β-actin as loading control; n = 1. (**B**,**C**) MSC pellets were cultured in the absence (CTRL) or presence of LDN-21 starting at day 0 or IWP-2 from day 14 on and alkaline phosphatase (ALP) enzyme activity was determined at weekly intervals in culture supernatants pooled from four pellets. Control pellets received the corresponding amount of DMSO solvent (0.02% for LDN-21, 0.04% for IWP-2). Experiments were performed with three independent MSC donor populations (n = 3). Means ± SEM are shown. *$p \leq 0.05$, **$p \leq 0.01$ according to LSD-corrected ANOVA.

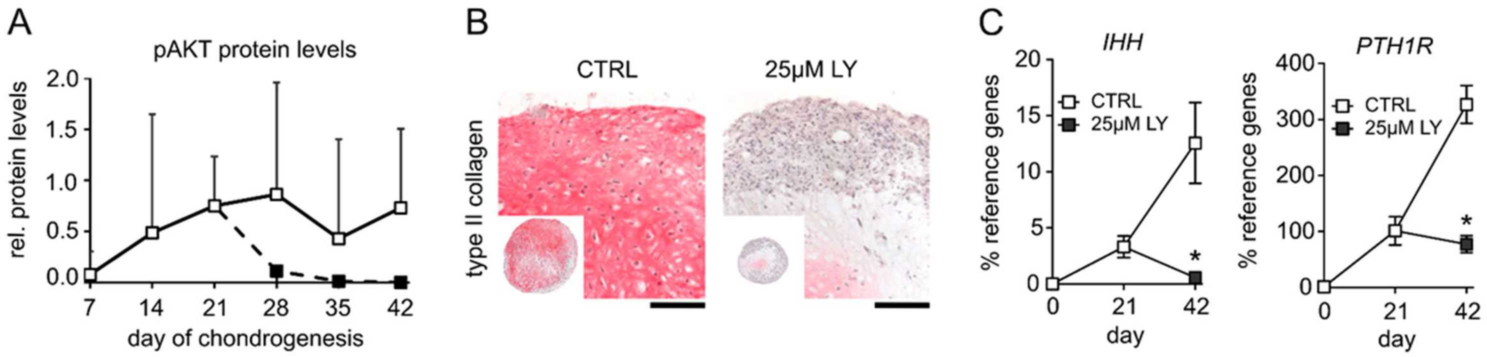

Supplemental Figure S2: AKT inhibition impairs MSC chondrogenesis. MSC pellets were subjected to chondrogenic culture for 6 weeks and treated with 25µM LY or 0.1% DMSO solvent (CTRL) starting at day 21 of chondrogenesis. (**A**) Densitometric analysis of Western blots shown in Figure 3A. Protein levels of pAKT at indicated time points were referred to β-actin. Medians ± 95% confidence intervals are shown; n = 3. (**B**) Paraffin sections of day 42 MSC pellets were stained for type II collagen by immunohistochemistry. Scale bars: 100 µm for enlarged sections and 1mm for insets. (**C**) Gene expression analysis of *IHH* and *PTH1R* by qPCR using *CPSF6* and *RPL13* as reference genes. Experiments were performed in three independent MSC donor populations. Graphs show means ± SEM. *$p \leq 0.05$ compared to CTRL at the same time point, MWU.

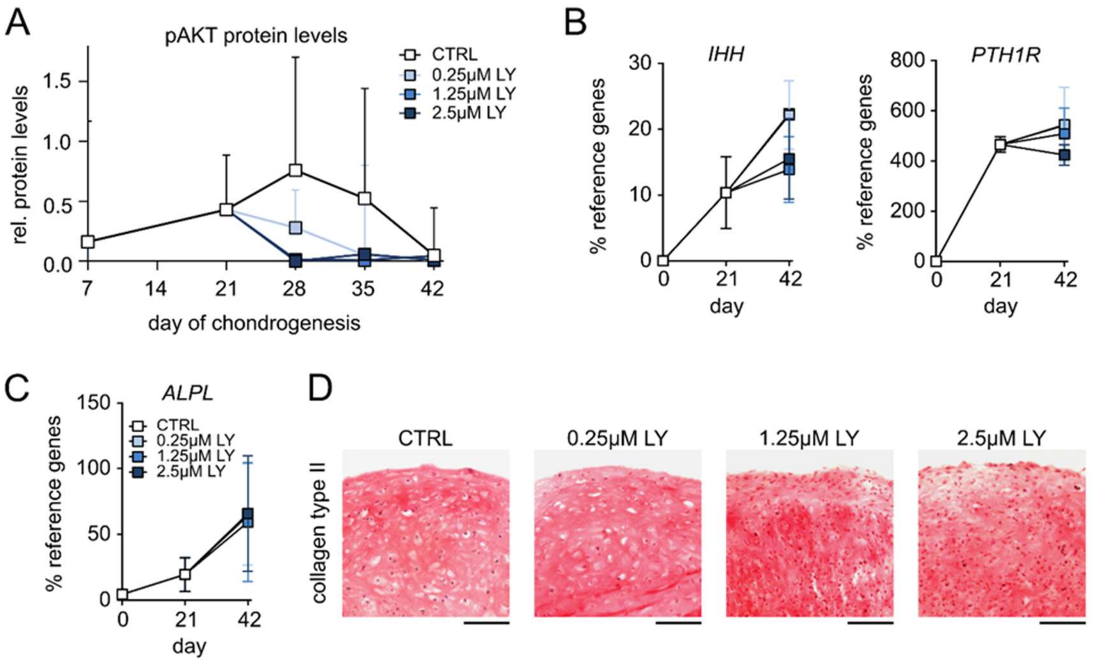

Supplemental Figure S3: Dose-dependent effects of PI3K/AKT inhibition on gene expression of *IHH*, *PTH1R* and *ALPL* as well as type II collagen deposition. MSC pellets were subjected to chondrogenic culture for 6 weeks and treated with 0.25 µM, 1.25 µM or 2.5 µM LY294002 (LY) or 0.01% DMSO solvent (CTRL) starting at day 21 of chondrogenesis. (**A**) Densitometric analysis of Western blots shown in Figure 4A. Protein levels of pAKT at indicated time points were referred to β-actin. Medians ± 95% confidence intervals are shown; n = 3. (**B,C**) Gene expression analysis by qPCR for the hypertrophic markers *IHH* and *PTH1R* and for the osteogenic marker *ALPL* using *CPSF6* and *RPL13* as reference genes. (**D**) Paraffin sections of day 42 MSC pellets were stained by immunohistochemistry to visualize type II collagen. Scale bars: 100µm. Shown is one representative of three independent experiments with three independent MSC donor populations (n = 3). Graphs show means ± SEM.

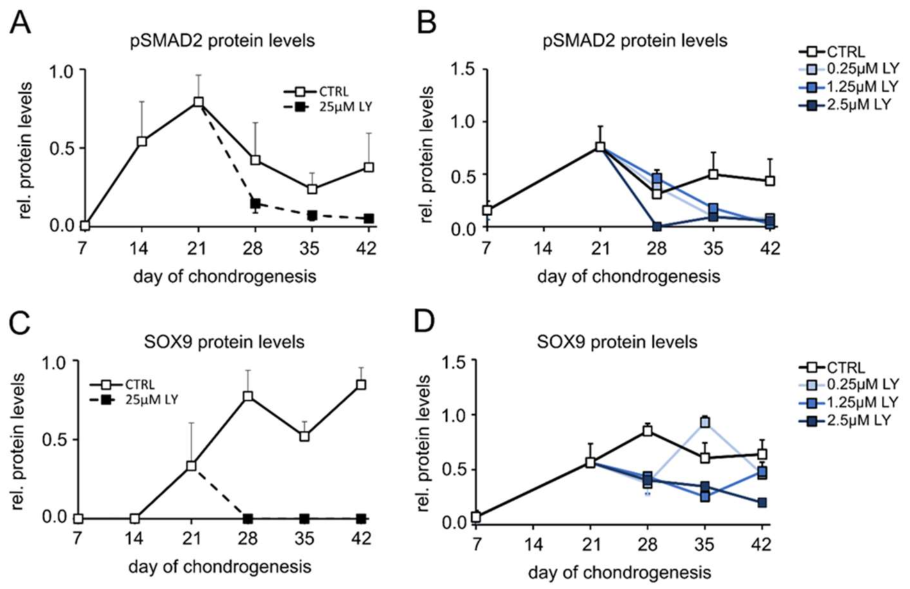

Supplemental Figure S4: PI3K/AKT signaling is important for TGFβ activity and SOX9 protein accumulation. Densitometric analysis of Western blots shown in Figure 5A-D. The effect of 25µM (A, C; n = 2–3) or 0.25-2.5 µM LY (B, D; n = 3) from day 21 to 42 on protein levels of pSMAD2 (**A**,**C**) or SOX9 (**B**,**D**) was investigated at indicated time points. β-actin was used as loading control. Means ± SEM are shown.

Supplemental Table S1: Primer pairs for qPCR in alphabetical order.

Gene	Forward	Reverse
ACAN	5´-GGAACCACTTGGGTCACG-3´	5´-GCACATGCCTTCTGCTT-3´
ALPL	5´-CACCAACGTGGCTAAGAATG-3´	5´-ATCTCCAGCCTGGTCTCCTC-3´
COL10A1	5´-TTTACGCTGAACGATACCAAA-3´	5´-TTGCTCTCCTCTTACTGCTAT-3´
COL2A1	5´-TGG CCT GAG ACA GCA TGA C-3´	5´-AGTGTTGGGAGCCAGATTGT-3´
CPSF6	5´-AAGATTGCCTTCATGGAATTGAG-3´	5´-TCGTGATCTACTATGGTCCCTCTCT-3´
IBSP	5´-CAGGGCAGTAGTGACTCATCC-3´	5´-TCGATTCTTCATTGTTTTCTCCT-3´
IHH	5´-CGACCGCAATAAGTATGGAC-3´	5´-GGT GAG CGG GTG TGA GTG -3´
PTH1R	5´-GGTGAGGTGGTGGCTGT-3´	5´-AGCATGAAGGACAGGAAC-3´
RPL13	5´-CATTTCTGGCAATTTCTACAG-3´	5´-CAGGCAACGCATGAGGAAT-3´
